# Racial Differences in Opiate Administration for Pain Relief at an Academic Emergency Department

**DOI:** 10.5811/westjem.2015.3.23893

**Published:** 2015-04-21

**Authors:** R. Myles Dickason, Vijai Chauhan, Astha Mor, Erin Ibler, Sarah Kuehnle, Daren Mahoney, Eric Armbrecht, Preeti Dalawari

**Affiliations:** * New York Hospital Queens, Department of Emergency Medicine, Flushing, New York; † Saint Louis University School of Medicine, Division of Emergency Medicine, St. Louis, Missouri; ‡ St. Luke’s Roosevelt Hospital Center, Department of Surgery, New York, New York; § Maricopa Medical Center, Department of Emergency Medicine, Phoenix, Arizona; ¶ University of Nevada School of Medicine, Department of Emergency Medicine, Las Vegas, Nevada; || Saint Louis University Center for Outcomes Research, St. Louis, Missouri

## Abstract

**Introduction:**

The decision to treat pain in the emergency department (ED) is a complex, idiosyncratic process. Prior studies have shown that EDs undertreat pain. Several studies demonstrate an association between analgesia administration and race. This is the first Midwest single institution study to address the question of race and analgesia, in addition to examining the effects of both patient and physician characteristics on race-based disparities in analgesia administration.

**Methods:**

This was a retrospective chart review of patients presenting to an urban academic ED with an isolated diagnosis of back pain, migraine, or long bone fracture (LBF) from January 1, 2007 to December 31, 2011. Demographic and medication administration information was collected from patient charts by trained data collectors blinded to the hypothesis of the study. The primary outcome was the proportion of African-Americans who received analgesia and opiates, as compared to Caucasians, using Pearson’s chi-squared test. We developed a multiple logistic regression model to identify which physician and patient characteristics correlated with increased opiate administration.

**Results:**

Of the 2,461 patients meeting inclusion criteria, 57% were African-American and 30% Caucasian (n=2136). There was no statistically significant racial difference in the administration of any analgesia (back pain: 86% vs. 86%, p=0.81; migraine: 83% vs. 73%, p=0.09; LBF: 94% vs. 90%, p=0.17), or in opiate administration for migraine or LBF. African-Americans who presented with back pain were less likely to receive an opiate than Caucasians (50% vs. 72%, p<0.001). Secondary outcomes showed that higher acuity, older age, physician training in emergency medicine, and male physicians were positively associated with opiate administration. Neither race nor gender patient-physician congruency correlated with opiate administration.

**Conclusion:**

No race-based disparity in overall analgesia administration was noted for all three conditions: LBF, migraine, and back pain at this institution. A race-based disparity in the likelihood of receiving opiate analgesia for back pain was observed in this ED. The etiology of this is likely multifactorial, but understanding physician and patient characteristics of institutions may help to decrease the disparity by raising awareness of practice patterns and can provide the basis for quality improvement projects.

## INTRODUCTION

Analgesia administration in the emergency department (ED) involves complex decisions based on multiple conscious and subconscious factors. Disparities in healthcare are propagated by subconscious stereotypical beliefs about patients (implicit bias), the patient-physician interaction, and patient factors including attitude, intention, self-efficacy, and disclosure.^1^ One hypothetical model proposes that provider interpersonal behaviors such as warmth, question-asking style, and patient-physician participatory style may influence patient cognitive factors such as their attitudes towards their care, physician, and encounter, disclosure of pertinent social and medical information, behavioral intentions during the encounter, and autonomy in taking action (self-efficacy).^2^ Other factors such as the reported level of pain and the availability of objective evidence of injury also contribute to physician beliefs and actions.^3^,^4^ Complicating the decision to prescribe or administer opiate analgesia are the dual concerns of oligoanalgesia, and the rising abuse of prescription narcotic medications.

A review of the literature found several studies that demonstrated a racial disparity in analgesia and opiate administration in the ED.^5^–^8^ The first of these studies, published by Todd et al.^7^ in 1993, found that Hispanics with isolated long bone fractures (LBF) were twice as likely as non-Hispanic whites to receive no pain medication at their academic institution, which was not explained by patient language, intoxication, or injury severity. The same author, practicing at a different academic institution in 2000, found African-American patients with LBF were less likely than Caucasians to receive analgesia, even with similar pain scores.^8^

In contrast, other studies have failed to show a racial disparity in analgesia administration for LBF.^9^–^12^ Such disparate findings in the literature suggest that a correlation between race and analgesia may, to a degree, be attributable to institutional or regional variation throughout the country or different study methodologies.^9^ It is plausible that increased attention to the Agency for Healthcare Research and Quality’s (AHRQ) annual National Healthcare Disparities Report has led to narrowing the racial gap of analgesia administration for LBF, a relatively objective finding, over time.^13^ There is also a growing body of literature suggesting that physician characteristics and possibly the interplay between physician and patient characteristics may impact the administration of analgesia in the ED.^14^–^16^

The purpose of this study was to assess ED analgesia-prescribing habits on both a department-wide and physician level at an academic institution. This study examines patient and physician characteristics, including patient race that may play a role in a physician’s decision to administer analgesia. Three diagnoses (i.e., back pain, LBF, migraine) that have varying degrees of objective sources of pain are included for comparison purposes.

## METHODS

### Study Design and Setting

This was a retrospective chart review of patients seen between January 1, 2007, and December 31, 2011, at the adult ED of an urban university hospital in a Midwestern metropolitan area. The ED has 22 beds and the annual census ranged from 34,018 in 2007 to 37,362 in 2011 of which 64% were African-American and 51% were male. Forty-eight percent of our population was on Medicaid or was uninsured. The 30 physicians in this group were comprised of 16 full-time and 14 part-time attending physicians who practiced in the ED during this timeframe. All full-time attending physicians were trained in emergency medicine (EM), while some part-time attending physicians were trained in other medical specialties (e.g., internal medicine). The ED provides training for its EM residency program as well as rotations for non-EM specialties including internal medicine, psychiatry, anesthesia, orthopedics, and otolaryngology. Attending physicians are involved in every case and closely supervise residents. First-year residents (interns) must first discuss the patient and plan with the attending, prior to writing orders. Second- and third-year residents are allowed to write orders, including narcotic medications, prior to discussing patients with the attending. At the time of this study, there was no nurse-run pain protocol at triage. The university’s institutional review board approved this study.

### Selection of Participants

We used the International Classification of Diseases (ICD-9) codes to generate a list of medical record numbers and demographic information of all patients who presented to the ED with one of the following diagnoses: back pain or strain (ICD-9 724), migraine (ICD-9 346), or fracture of the humerus, femur, or tibia or fibula, (ICD-9 812, 821, 823, respectively; herein referred to as LBF). Inclusion criteria required participants to be aged 18 or older. We excluded patients with more than one fracture (such as humerus and tibia fracture) or multiple diagnoses.

### Measurements

We collected data following the guidelines of Gilbert and Lowenstein et al. including trained data collectors, standard data collection sheets, and inter rater reliability.^17^ Data collectors reviewed each patient’s chart and recorded whether the patient received any analgesics by the physician or resident, and if so, whether the patient received an opiate of any dose. Other demographic information collected from the chart was triage acuity on a scale of 1–4 (where one was the highest acuity and four the least acute level), race, age and gender**.** Collectors were blinded to the hypothesis of the study. Their training included instruction on the structure of the electronic medical record, the definition of the study variables, and the data collection procedure. We performed an audit of each data collector’s results based on a randomly selected set of 10 charts to ensure the precision of each collector’s measurements and the interpersonal reliability of the results. In all, 40 charts were reviewed, 10 from each data collector. There was perfect agreement between the information gathered by the collectors and the auditor’s samples.

A patient was considered to have received analgesia if an analgesic drug was administered in the ED or prescribed at discharge. The list of medications considered analgesia, those considered an opiate, and several drugs that notably were not considered analgesia, is presented below (Table 1).

### Outcomes

The primary objective was to compare the proportion of African-Americans who received analgesia (and if so, those who received an opiate) to Caucasians, for each diagnosis. A secondary analysis measured the modifying effect of patient and/or physician characteristics on the administration of analgesia. The following attributes of the attending physician who was responsible for the patient’s care were examined: gender, congruence by race and gender (i.e., patient and physician had the same race or same gender), completion of an EM residency.

### Analysis

We used a Pearson’s chi-squared test to compare baseline demographic characteristics between Caucasians and African-Americans, the proportion of patients receiving any type of analgesia by race, and the proportion of patients who received an opiate by race among patients who had received some form of analgesia.

A multiple logistic regression model was created to identify patient and physician characteristics associated with opiate administration for the only diagnosis in which a racial disparity was observed. First, we employed a univariate analysis to estimate the odds ratios for each variable independently. The construction of a multiple logistic regression model then followed a two-step process in which we assessed statistically significant variables for inclusion in a final model based on how their inclusion affected the odds ratio for Caucasians with back pain to receive an opiate, as compared to African-Americans. Variables with no significant impact were excluded. In this way, the final multiple logistic regression model represents the most parsimonious model for estimating the effect of race on opiate administration, adjusting for likely confounders.

In addition, physician level differences in opiate prescribing for Caucasians and African-Americans were calculated for those who treated at least 25 patients during the study period. We ranked physicians according to opiate-prescribing differences to illustrate the range of prescribing practice, but we made no adjustments for any patient-level attributes, such as acuity. Reporting of this descriptive analysis was limited to the diagnosis group(s) for which disparities were found.

We performed all statistical tests using SPSS version 18.0 (Chicago, IL) with the assistance of an independent statistical consultant. Statistical significance was defined using a two-tailed test with an alpha level of p<0.05. A Bonferroni adjustment ws applied to the interpretation of the study’s primary analysis regarding proportion of patients who received an opiate by two racial groups for three diagnoses; the adjusted alpha was 0.008.

## RESULTS

Of the 2,461 patients who met inclusion criteria, 741 (30.0%) were Caucasians, 1,395 (56.7%) African-Americans, 48 (2.0%) with race listed as “other,” and 277 (11.3%) patients with race listed as “unknown.” Baseline characteristics by diagnosis are compared in Table 2.

### Primary Results

Of the 2,461 patients who met inclusion criteria, we analyzed a total of 2,136 patients: 1,850 (75.2%) cases of back pain, 238 (9.7%) cases of migraine, and 373 (15.2%) cases of LBF. Patients (n=325) with other or unknown race were excluded from analysis (13%) (Table 2).

There was no statistically significant difference between the proportion of African-Americans and Caucasians who received some form of analgesia for any of the three diagnoses. Of patients who received analgesia and were diagnosed with a migraine or LBF, there was no statistically significant race-based difference in the likelihood of receiving an opiate. However, among patients who were diagnosed with back pain and received analgesia, African-Americans were less likely to receive an opiate than Caucasians (Table 3a and Table 3b): 50% versus 72%, p<0.001.

### Secondary Results

Between 2007 and 2011, the 1,850 patients presenting with back pain were seen by 30 attending physicians (Table 4). The number of patients seen by each physician varied greatly from 1 to 226. To understand if the race-based disparity in opiate administration for back pain reflected department-wide prescribing practices or whether this finding stemmed from one or two outlying physicians, we conducted an analysis of analgesia administration using 17 out of 30 physicians who saw more than 25 patients with back pain. Together, these 17 physicians saw 95% of the 1,850 patients (Table 5). The difference in opiate administration to African-Americans and Caucasians for each physician ranged from −9% to 50% (mean = 21%, standard deviation = 14%). Two physicians were statistical outliers, as defined as falling greater than 2.5 standard deviations from the mean—one prescribing more frequently to African-Americans, the other more frequently to Caucasians. Only one physician administered opiates more frequently to African-Americans; the other 16 physicians administered opiates more frequently to Caucasians.

Since univariate analysis showed that gender congruence was not a significant predictor of opiate administration it was removed from further development of the logistic regression model. Among patients presenting with back pain who received some form of analgesia, the final multivariate model estimated the odds ratio that a Caucasian patient would receive an opiate to be 2.41 (95% CI [1.67,3.46]), as compared to an African-American patient. This model controlled for acuity, age, physician gender, race congruence, and physician training in EM. This model revealed that higher acuity, older age, physician training in EM, and male physicians were positively associated with opiate administration (Table 6). Given the cohort design and 43.5% prevalence of non-opiate use, the odds ratio was converted to a risk ratio of 1.49 per calculation procedures described by Zhang and Yu (1998).^18^

## DISCUSSION

Racial and ethnic differences in pain management have been noted in many medical care settings (emergency departments, primary care offices), and for various types of pain (postoperative, nonmalignant, chronic, and cancer pain).^4^ The complex factors that mediate this disparity through the range of clinical settings have not clearly been elucidated, but research suggests that patient, provider, systemic, and cultural issues directly and interactively are involved.^3^,^4^,^19^ Other factors contributing to these disparities may include lack of education and quality improvement projects on pain management, and general reluctance to use or prescribe opiates.^16^,^20^ Our current study reflects the sentiments of Tait et al.^3^ that racial/ethnic disparities have proved difficult to change, despite the national focus on oligoanalgesia and decades of research on healthcare disparities.

In fact, we found similar results to a study of the National Hospital Ambulatory Medical Care Survey (NHAMCS) from 15 years ago. African-Americans (AA) and Caucasians received some form of analgesia at similar rates across all diagnoses (back pain, migraine, LBF).^6^ However, AA were less likely to receive opiates for back pain and migraine conditions, compared to Caucasians. Mills et al.^21^ studied a cohort of patients with undifferentiated abdominal pain and back pain (based on chief complaints) and found that white patients were 10% more likely to receive opiates than non-whites. Pletcher et al.^5^ (using NHAMCS 1993–2005) also found that overall analgesia administration (opiates and non-opioids) did not differ between non-whites and whites, but that opiates for various painful conditions, including back pain, were prescribed more frequently to whites (48% whites vs. 36% non-whites), even after controlling for covariates. They also noted that the differential in opiate prescribing by race/ethnicity did not decrease over time.^5^ Thus, while the disparity in any analgesia administration has decreased over time, our study suggests that a disparity in the type of analgesia may still exist. As noted by Tamayo-Sarver,^6^ the difference in opiate administration is noted in those with diagnoses that may have limited objective findings because the decision to administer an opiate “requires more trust of the patient by the physician.” This observation can account for the fact that opiate use for LBF was consistently higher than the other two diagnoses, irrespective of race. Objective findings in LBF include radiographic evidence of fracture, visible deformity, or broken skin/bone visible on physical examination. In contrast, migraines and back pain commonly have limited objective findings on physical examination and are more subjective sources of pain. Studies also suggest that minority race/ethnicity was associated with lower rates of opiate prescriptions for discharged patients.^14^,^22^

Patient-physician communication and assessment of pain by the provider are important factors in the treatment of pain. Numerous studies have related that physicians underestimate pain more in racial/ethnic minorities, especially for patients reporting high pain severity.^4^,^19^ Patient factors such as decreased satisfaction with patient-physician communication among African-American patients, and the decreased assertiveness shown by minority patients during interactions with physicians can hinder a patient’s ability to express the nature of an ailment or the severity of a pain.^14^,^23^ While race concordance may enhance pain communication^3^ or patient satisfaction,^24^ in our study, it did not translate into a higher chance of receiving opiates for back pain when other variables were accounted for. Heins et al.^1^ did not note any racial concordance on clinically significant pain intensity reduction in the ED. They found non-white physicians were more likely to achieve pain reduction than white physicians despite lower opiate administration, possibly due to other characteristics of the clinical encounter unrelated to the medication treatment.^1^

Implicit bias that relies on unconscious racial stereotypes may influence pain management decisions such as assessment and credibility of pain, as well as possible misuse of opiate prescriptions.^3^,^19^,^25^–^27^ Even the most well-intentioned individuals, such as emergency physicians who provide the “safety net” of healthcare, can lean towards unconscious stereotypes when fatigued or required to make quick decisions with little information (i.e. in cases of clinical uncertainty and the fast-paced environment of the ED).^28^ A clinical vignette study on chronic low back pain suggests that prescribing practices vary, not only by physician gender, but by cognitive load in which greater load may enhance the implicit biases one may have.^27^ Under high cognitive load, male physicians were more likely to prescribe opioids to white male patients. Under low cognitive load, male physicians were more likely to prescribe opioids for black male patients, implying the male physicians were able to correct for their inherent bias when there was more time and resources to do so. Surprisingly and inexplicably, the pattern for female physicians was reversed.^27^ Safdar et al.^16^ found gender congruence in the administration of opiates in the ED, which may be partially explained by experimental studies that have found men report higher pain tolerance and lower pain intensity when reported to a female.^16^,^19^ We found no such association between gender congruence and opiate administration in our patient population. The wide range of results on patient and provider characteristics in regards to analgesia administration and pain reduction makes regional and institutional data acquisition important in identifying practice patterns. This is especially true since many educational programs addressing racial disparities in healthcare focus on patient factors instead of provider factors (i.e. lack of trust).^29^ Indeed, much of ED literature on factors associated with racial healthcare disparities focus on improving access to care, as opposed to any physician factors, especially since this can be difficult and sensitive to examine.^24^

Regarding approaches to improve pain management in the ED, clinical education shows promise.^14^,^30^ Heins et al.^14^ found that EM-trained physicians, similar to our study, and EM physicians practicing for fewer than three years—i.e. physicians with more recent EM education—were more likely to administer opioids in the ED and ensure adequate analgesia. However, those experienced EM physicians were more likely to prescribe opioids at discharge.

Looking forward, the question remains on the best ways to mitigate racial disparities in the ED for pain management, and ED healthcare in a broader context. Programs that may help include empathy training, communicating about expectations of pain relief in the ED, and cross-cultural training.^3^,^26^ There is a dearth of evidence, however, that cross-cultural training actually reduces racial/ethnic healthcare disparities.^28^,^29^ Awareness of and learning about implicit bias and how it operates in clinical settings is needed in undergraduate, graduate, and postgraduate programs to improve racial disparities.^26^,^28^ Richardson et al.^31^ suggests quality improvement programs such as periodic retrospective review of ED physician data on points of known disparate care so that physicians can be more cognizant of any implicit bias. In addition, a multidisciplinary approach using techniques from social and psychological science research could help to inform clinical educators and practicing physicians on the nuances of stereotypes and ways to overcome implicit bias.

## LIMITATIONS

Potential limitations include those associated with retrospective chart reviews and the single-institution study design that limits the generalizability of the results. Self-identification of race, perception of others’ race, and race relations differ regionally; accordingly, it is impossible to say whether the findings of the present study reflect only regional patterns, or if they are applicable to other areas of the country. Eleven percent of the study population had no recorded race documented in the medical chart (and 2% with “other” race) were excluded from analysis. It is unknown whether race, as recorded in the chart, was self-identified or assumed. Ethnicity (i.e. Hispanic white versus non-Hispanic white) was not elucidated from race although other studies have shown that ethnic minorities are also less likely to receive opiates.^3^,^4^,^6^ In actuality race itself is not necessarily homogenous and cultural differences within the group may confound differences (African patients and African-American lumped into one group).^32^

Another limitation involves the use of diagnostic codes, as opposed to chief complaints, as inclusion criteria. Many conditions can cause back pain and the exact etiology cannot always be elucidated within one ED visit. Thus a diagnostic code of unspecified back pain may in fact be sciatica, nephrolithiasis, or an epidural abscess (that was not diagnosed in the ED), and be misclassified, although it is doubtful that there would be a differential by race. Along similar lines, selection bias may explain why there was no race-based disparity in opiate administration for migraines. It is plausible that only patients with a previous documented diagnosis of migraine or migraine recorded in their past medical history received this code. Thus, the complaint of pain may have been considered to be more objective evidence (compared to complaints of headaches of unclear etiology) due to their history.

Finally, the retrospective review of medical records allows only a limited understanding of clinical decision-making. Factors that are not always captured in patients’ charts may have had a bearing on the decision to administer analgesia; these include nonverbal communication, verbal communication (asking to receive or not receive opiates), socioeconomic status, and education level. Other factors that may have impacted reception of opiates include mode of arrival, duration of pain, intoxication, and pain score. Pain scores can be extremely subjective with wide variability between patients and may not be able to discriminate between those who want pain medication and those who do not.^33^,^34^ Analgesia was defined as in Table 1 with notable exclusions of muscle relaxers and neuroactive agents that can also be administered for pain relief. Similarly, sumatriptan, caffeine, or anti-nausea medications such as prochlorperazine may have been used to relieve the pain of a migraine headache, but these drugs were also not considered analgesics.

The use of opiates for back pain and migraine conditions in this ED may seem high at 49% and 43%, especially in light of guidelines calling to limit opiate use for these conditions. The American Academy of Neurology guidelines from 2000 recommend opiate use only as a rescue medication after first-line therapy has failed, and the 2008 American College of Emergency Physicians clinical policy on headache makes no mention of opiate use.^35^ However, using NHACMS data from 2002 to 2006, Friedman et al.^36^ note that of medications administered or prescribed for low back pain, opiates were the most frequently used at 61.7% (compared to non-steroidal medications at 49.6%). Mazer-Amirshahi et al.^35^,^37^ note an overall increase in the ED administration and prescription of opiates in pain-related ED visits (including headache) from 2001 to 2010 with the Midwest region having a greater proportional increase and showing a greater increase in narcotic use than others. While it is unclear what is driving this trend, the authors’ theories include patient preferences, drug shortages, and patient satisfaction experiences.

Furthermore, this study was performed at a teaching hospital, where both residents and physicians were involved with care of the patient. Either member of the team could give analgesia; however, the physician level factors were attributed to the primary attending responsible for the patient.

## CONCLUSION

No race-based disparity in overall analgesia administration was noted for all three conditions: LBF, migraine, and back pain at this Midwestern institution. However, we observed a race-based disparity in the likelihood of receiving opiate analgesia for back pain in this ED with Caucasians 1.5 times more likely than African-Americans to receive opiate analgesia. The etiology of this is likely multifactorial, but understanding physician and patient characteristics of institutions may help to decrease the disparity by raising awareness of practice patterns and can provide the basis for quality improvement projects. Additionally, this study provides an approach for identifying department-wide race-based disparities, allowing institutions to determine if steps addressing potential bias should be focused on a department as a whole or on specific members. More interdisciplinary research is needed to address ways in which racial disparities related to pain management in the ED can be mitigated.

## Figures and Tables

**Table 1:** Non-opiate analgesia, opiate analgesia, and notable non-analgesia medications.

Non-opiate analgesia	Opiate analgesia	Non-analgesia
aspirin	tramadol	methocarbamol
ibuprofen	hydromorphone	cyclobenzaprine
naproxen	fentanyl	benzodiazepines
ketorolac	morphine	gabapentin
acetaminophen	hydrocodone	

**Table 2:** Baseline characteristics of study population by diagnosis, 2007–2011, N=2,461.

	Caucasian	African-American	p-value*	Other race	p-value*	Unknown race	p-value*	Total
Back pain								
N	469	1099		32		250		1850
Age, median	40	40	0.43	47	0.59	42	<0.01	41
Male (%)	277 (59%)	514 (47%)	<0.01	18 (56%)	0.75	138(55%)	0.31	947 (51%)
Acuity**	3.2	3.5	<0.01	3.6	<0.01	3.5	<0.01	3.4
Time in ED (h)	2.2	3.4	0.13	3.6	0.32	3.1	0.32	3.4
Migraine								
N	83	133		6		16		238
Age, median	34	36	0.72	36	0.45	31	0.17	34
Male (%)	20 (24%)	24 (18%)	0.31	1 (17%)	0.56	3 (19%)	0.76	48 (20%)
Acuity**	2.8	3.0	0.03	3.2	0.28	3.3	<0.01	2.9
Time in ED (h)	5.0	4.6	0.39	5.3	0.70	3.1	0.02	4.6
Long bone fracture								
N	189	163		10		11		373
Age, median	50	36	<0.01	26	0.04	47	0.05	45
Male (%)	105 (56%)	102 (63%)	0.18	10 (100%)	0.01	7 (64%)	0.76	225 (60%)
Acuity**	2.5	2.7	0.06	2.9	0.47	3.1	0.08	2.7
Time in ED (h)	4.6	4.3	0.69	4.6	0.85	5.3	0.77	4.3

*ED,* emergency department

* As compared to Caucasians (h)=hours.

** Range=1–4, 1=highest acuity. Acuity listed is the mean.

**Table 3a:** Percent of patients receiving any analgesia by diagnosis and race.

	Back pain			Migraine			Long bone fracture		
	
Analgesia	n	% Analgesia	p-value	n	% Analgesia	p-value	n	% Analgesia	p-value
Caucasian	469	86%	0.81	83	73%	0.09	189	90%	0.17
African-American	1099	86%		133	83%		163	94%	
Total	1568			216			352		

**Table 3b:** Percent of patients receiving opiate by diagnosis and race.

	Back pain			Migraine			Long bone fracture		
	
Opiate	n	% Opiate	p-value	n	% Opiate	p-value	n	% Opiate	p-value
Caucasian	403	72%	<0.001	61	62%	0.11	170	98%	0.49
African-American	949	50%		111	49%		154	97%	
Total	1352			172			324		

**Table 4:** Attending physician characteristics (n=30).

Race	n	%
Caucasian	25	83%
African-American	2	7%
Other	3	10%
Gender		
Male	20	67%
Female	10	33%
Emergency medicine trained		
Yes	19	63%
No	11	37%
Total	30	

**Table 5:** Treating practices of physicians who had seen 25 or more patients with back pain.

		Percentage of patients with back pain administered opiates		
			
Physician	% Volume of patients with back pain**	African-American	Caucasian	% Difference between African-American and Caucasian*
1	2	59	50	−9
2	7	79	81	1
3	6	46	50	4
4	4	60	67	6
5	2	56	67	11
6	7	35	48	13
7	4	37	53	16
8	10	63	80	17
9	7	26	48	22
10	5	58	80	23
11	8	58	84	26
12	8	49	76	27
13	5	55	85	30
14	8	39	74	35
15	4	39	75	36
16	5	45	81	37
17	2	50	100	50
Mean ± SD		50±13	71±16	21±14
Median		50	75	22

* Numbers may not sum to 100% due to rounding.

** 5% of patients were seen by 13 physicians who saw fewer than 25 study patients each with back pain. These patients were not included in this analysis.

**Table 6:** Univariate and multiple logistic regression analysis of opiate administration to patients with back pain who received analgesia.

	Univariate analysis n=1352		Multivariable model n=1262	
	
Characteristic	OR	95% CI	OR	95% CI
Patient				
Race (Caucasian)	2.55	(1.97, 3.27)*	2.41	(1.67, 3.46)*
Sex (female)	0.84	(0.67, 1.04)		
Age	1.03	(1.02, 1.03)*	1.03	(1.02, 1.04)*
Acuity$	0.41	(0.33, 0.50)*	0.49	(0.40, 0.60)*
Physician				
Sex (female)	0.78	(0.55, 0.94)*	0.72	(0.55, 0.93)*
Same race as patient	1.70	(1.32, 2.18)*	0.94	(0.65,1.35)
Same gender as patient	0.94	(0.76, 1.17)		
Trained in emergency medicine%	1.53	(1.19, 1.97)*	1.45	(1.09, 1.93)*

* Statistically significant p<0.05.

$ n=1264.

% n=1350.
